# Amount of ascites impacts survival in patients with hepatocellular carcinoma undergoing transarterial chemoembolization advocating for volumetric assessment

**DOI:** 10.1038/s41598-024-67312-2

**Published:** 2024-07-17

**Authors:** Lukas Müller, Daniel Bender, Simon J. Gairing, Friedrich Foerster, Arndt Weinmann, Jens Mittler, Fabian Stoehr, Moritz C. Halfmann, Aline Mähringer-Kunz, Peter R. Galle, Roman Kloeckner, Felix Hahn

**Affiliations:** 1 Department of Diagnostic and Interventional Radiology, University Medical Center of the Johannes Gutenberg University Mainz, Langenbeckstr. 1, 55131 Mainz, Germany; 2 Department of Internal Medicine I, University Medical Center of the Johannes Gutenberg University Mainz, Mainz, Germany; 3 Department of General, Visceral and Transplant Surgery, University Medical Center of the Johannes Gutenberg University Mainz, Mainz, Germany; 4 Institute of Interventional Radiology, University Hospital Schleswig-Holstein-Campus Luebeck, Luebeck, Germany

**Keywords:** Hepatocellular carcinoma, Prognosis, Therapeutic chemoembolization, Portal hypertension, Ascites, Cancer imaging, Cancer therapy

## Abstract

Preliminary work has shown that portal hypertension plays a key role for the prognosis in patients with hepatocellular carcinoma (HCC) undergoing transarterial chemoembolization (TACE). Specifically, the presence of ascites appears to be a strong negative predictor for these patients. However, it remains unclear whether different ascites volumes influence prognosis. Therefore, the aim of this work was to investigate the influence of different ascites volumes on survival for patients with HCC undergoing TACE. A total of 327 treatment-naïve patients with HCC undergoing initial TACE at our tertiary care center between 2010 and 2020 were included. In patients with ascites, the fluid was segmented, and the volume quantified by slice-wise addition using contrast-enhanced CT imaging. Median overall survival (OS) was calculated and univariate and multivariate Cox regression analysis has been performed. Ascites was present in 102 (31.9%) patients. Ascites volume as continuous variable was significantly associated with an increased hazard ratio in univariate analysis (*p* < 0.001) and remained an independent predictor of impaired median OS in multivariate analysis (*p* < 0.001). Median OS without ascites was 17.1 months, and therefore significantly longer than in patients with ascites (6.4 months, *p* < 0.001). When subdivided into groups of low and high ascites volume in relation to the median ascites volume, patients with low ascites volume had a significantly longer median OS (8.6 vs 3.6 months, *p* < 0.001). Ascites in patients with HCC undergoing TACE is strongly associated with a poor prognosis. Our results show that not only the presence but also the amount of ascites is highly relevant. Therefore, true ascites volume as opportunistic quantitative biomarker is likely to impact clinical decision-making once automated solutions become available.

## Introduction

Hepatocellular carcinoma (HCC) is among the most common cancer entities, and it ranks second among diseases responsible for cancer-related deaths ^1,2^. The European Association for the Study of the Liver (EASL) and the American Association for the Study of Liver Diseases (AASLD) guidelines have recommended the Barcelona Clinic Liver Cancer (BCLC) classification system as framework for patient stratification, treatment allocation, and prognosis prediction ^3,4^. According to the BCLC classification, transarterial chemoembolization (TACE) is recommended for patients with intermediate stage HCC ^5^. Additionally, in the concept of stage migration and individual treatment decision-making, TACE is also applied in other BCLC stages and ranks among the most commonly applied treatments for HCC worldwide ^6^.

A large amount of patients develop HCC as a consequence of liver cirrhosis ^3^. Liver cirrhosis causes progressive changes in the splanchnic circulation, which lead to an increase in portal pressure ^7^. In addition to portal hypertension, many patients with liver cirrhosis suffer from a retention of sodium and water ^8^. In combination, both factors lead to the development of ascites, which has a negative impact on the prognosis of these patients ^8^. Factors associated with portal hypertension have also been identified as highly relevant to prognosis in patients with HCC ^9^. In particular, the presence of ascites has a major influence on the prognosis of patients with HCC undergoing TACE ^10^. However, this does not take into account the different amounts of ascites: While some patients only have a narrow seam of ascites around the liver or spleen, other patients have large amounts of ascites that fill the entire abdominal cavity.

**Figure 1:**
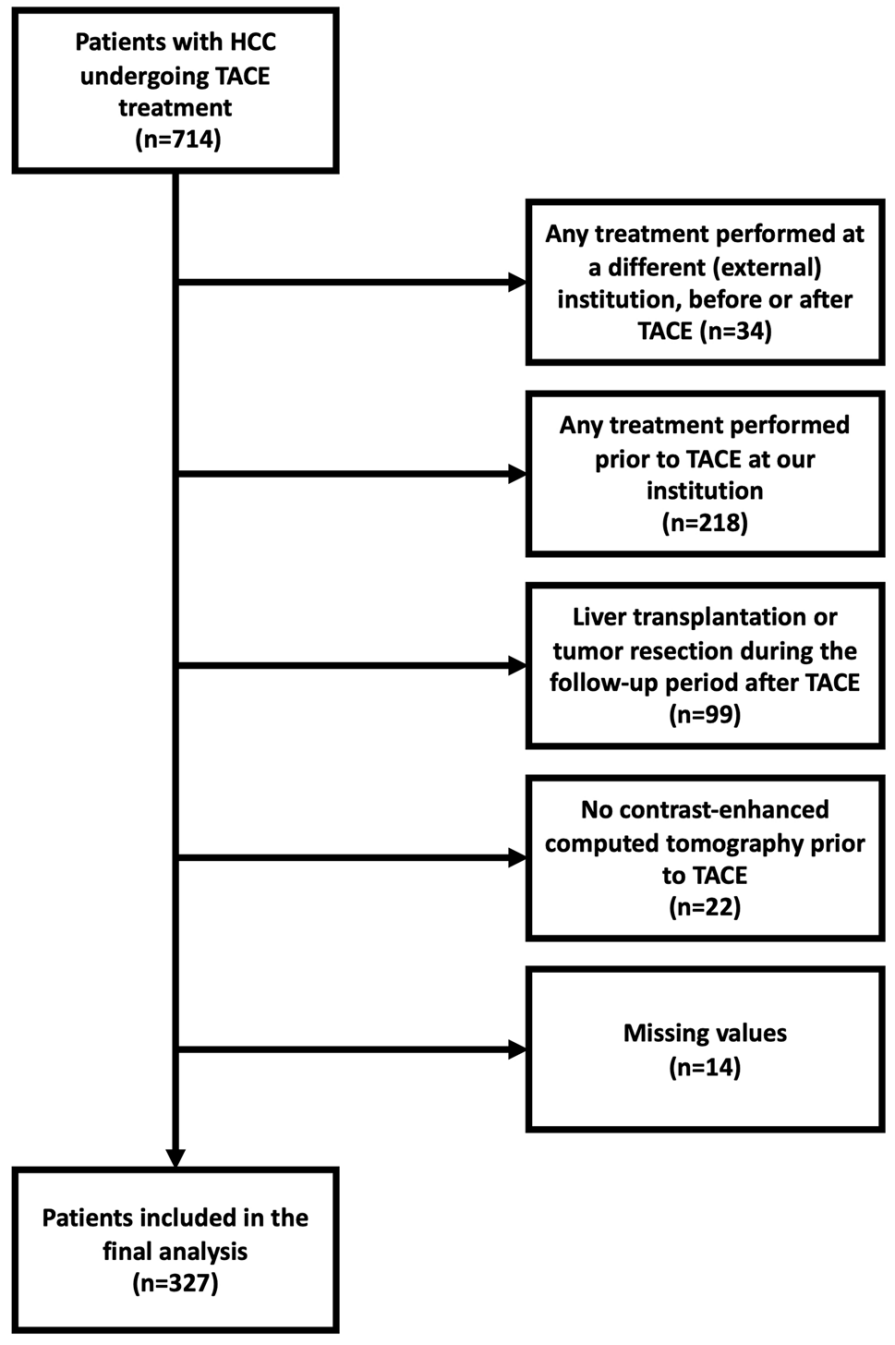
Flowchart of the patient selection process for this study.

To date, it is unclear what role the amount of ascites plays in the prognosis of patients. Our hypothesis was that besides the presence of ascites itself, the amount of ascites also plays a critical prognostic role. Therefore, the aim of this study was to investigate the prognostic role of precise ascites volume in patients with HCC undergoing TACE (Fig. 1).

## Results

### Baseline

Of the 327 patients, 51 (15.6%) were female and the median age was 69 (IQR 63–75) years. A total of n = 102 patients (31.2%) had ascites in their CT scans prior to TACE. Baseline characteristics of the included patients are depicted in Table 1.

**Table 1:** Baseline patient characteristics.

Variable	All patients (n = 327)
Median age, years (IQR)	69 (63–75)
Sex, n (%)	
Female	51 (15.6)
Male	276 (84.4)
Etiology, n^a^	
Alcohol	156
Hepatitis C	55
Hepatitis B	28
NAFLD	26
Hemochromatosis	9
AIH/PBC/PSC	5
Unknown/Other	27
Child–Pugh stage, n (%)	
A	120 (36.7)
B	133 (40.7)
C	30 (9.2)
No cirrhosis	44 (13.4)
ALBI grade, n (%)	
1	17 (5.2)
2	207 (63.3)
3	103 (31.5)
BCLC stage, n (%)	
0	0
A	60 (18.3)
B	166 (50.8)
C	71 (21.7)
D	30 (9.2)
Tumor load	
Median tumor size, mm (IQR)	42 (28–64)
Tumor number, n (%)	
Unifocal	74 (22.6)
Multifocal	253 (77.4)
Blood	
Median albumin level, g/l (IQR)	31 (27–35)
Median bilirubin level, mg/dl (IQR)	1.4 (0.8–2.2)
Median platelet count, per nl (IQR)	129 (87–192)
Median AST level, U/l (IQR)	64 (46–100)
Median ALT level, U/l (IQR)	41 (28–61)
Median INR (IQR)	1.2 (1.1–1.3)
Median AFP level, ng/ml (IQR)	30 (7–767)
Presence of ascites, n (%)	
Yes	102 (31.2)
No	225 (68.8)
Median ascites, ml (IQR)	613.5 ml (IQR 111.8–1559.8 ml)
Subsequent treatment	
Yes^c^	72 (22.0)
No	255 (78.0)

^a^More than one etiology was possible for liver disease; thus, percentages were not calculated.

^b^In the subset of patients with ascites (n = 225). Abbreviations: NASH, nonalcoholic steatohepatitis; AIH, autoimmune hepatitis; PBC, primary biliary cholangitis; PSC, primary sclerosing cholangitis; BCLC, Barcelona Clinic Liver Cancer; AST, aspartate aminotransferase; ALT, alanine aminotransferase; AFP, alpha fetoprotein.

^c^Sorafenib (n = 33), lenvatinib (n = 13), selective internal radiation therapy (n = 12), atezolizumab in combination with bevacizumab (n = 6), pembrolizumab (n = 2), pembrolizumab in combination with regorafenib (n = 2), lenvatinib followed by sorafenib (n = 1), linifanib followed by sorafenib (n = 1), nivolumab (n = 1), ramucirumab (n = 1).

### Quantified ascites volume

**Figure 2:**
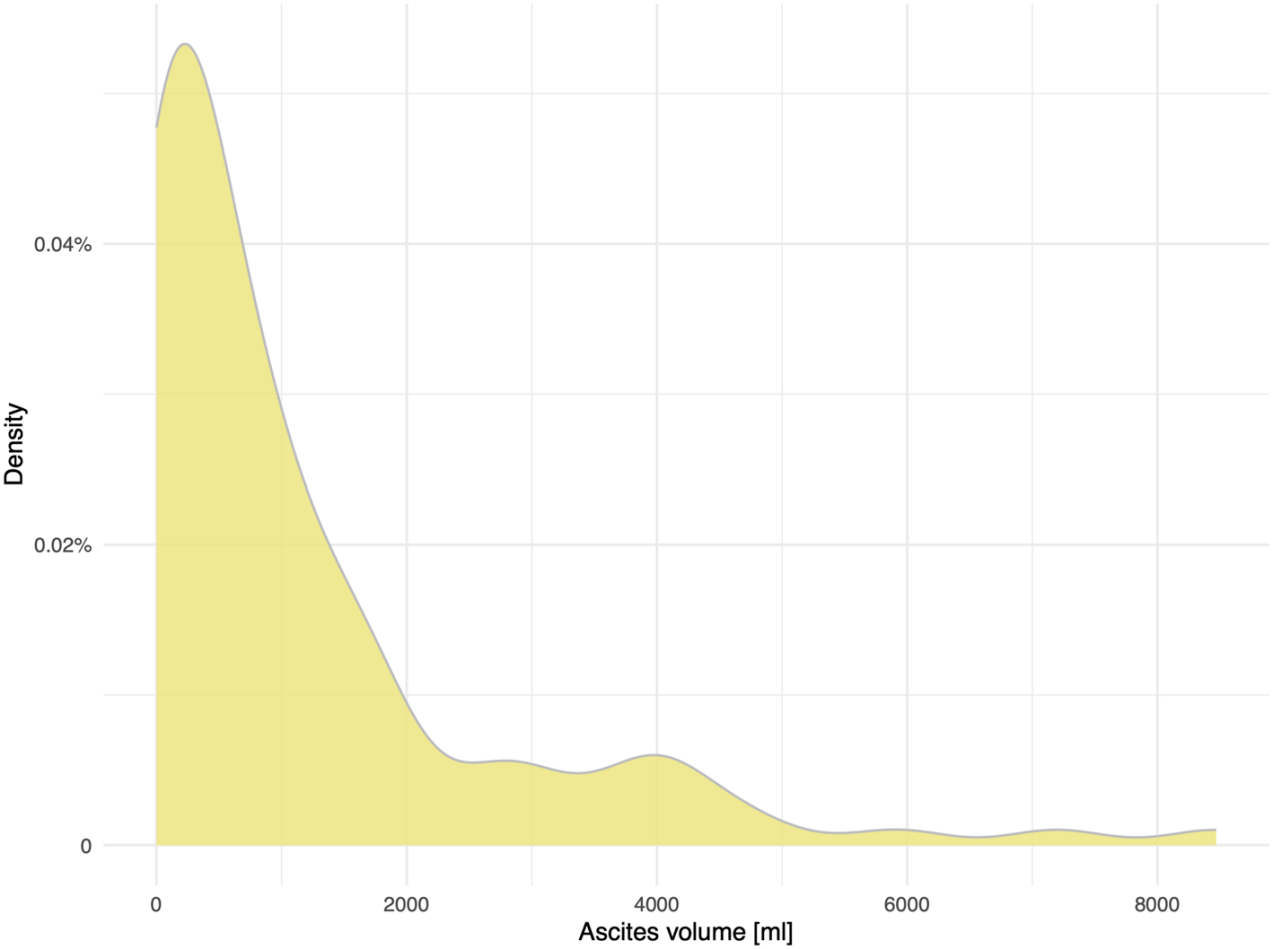
Distribution of the ascites volumes among all patients with ascites (n = 102).

Among patients with ascites, the median volume was 613.5 ml (IQR 111.8–1559.8 ml). The distribution of the ascites volume is shown in Fig. 2. Among the 102 patients with ascites, 39 (38.2%) patients were within BCLC stage C or D. Of those, nine patients had at least one extrahepatic metastasis. One of these patients had peritoneal metastases.

The ascites volume showed a significant correlation with albumin (moderate correlation) and bilirubin (weak correlation), while there was no significant correlation with the thrombocyte count, INR, the tumor diameter and the number of tumors (Table 2).

**Table 2:** Correlation between Ascites volume and surrogates of liver function and tumor burden.

Parameter	Correlation coefficient	*p* value
Liver function		
Albumin	− 0.36	< 0.001
Bilirubin	0.23	< 0.001
Thrombocytes	0.02	0.79
INR	0.00	1.00
Tumor burden		
Largest tumor diameter	0.01	0.83
Number of tumors	0.04	0.45

Correlation coefficient: ≤ 0.1, no correlation, > 0.1 weak correlation, > 0.3 moderate correlation, > 0.5 strong correlation.

### Survival analysis

**Figure 3:**
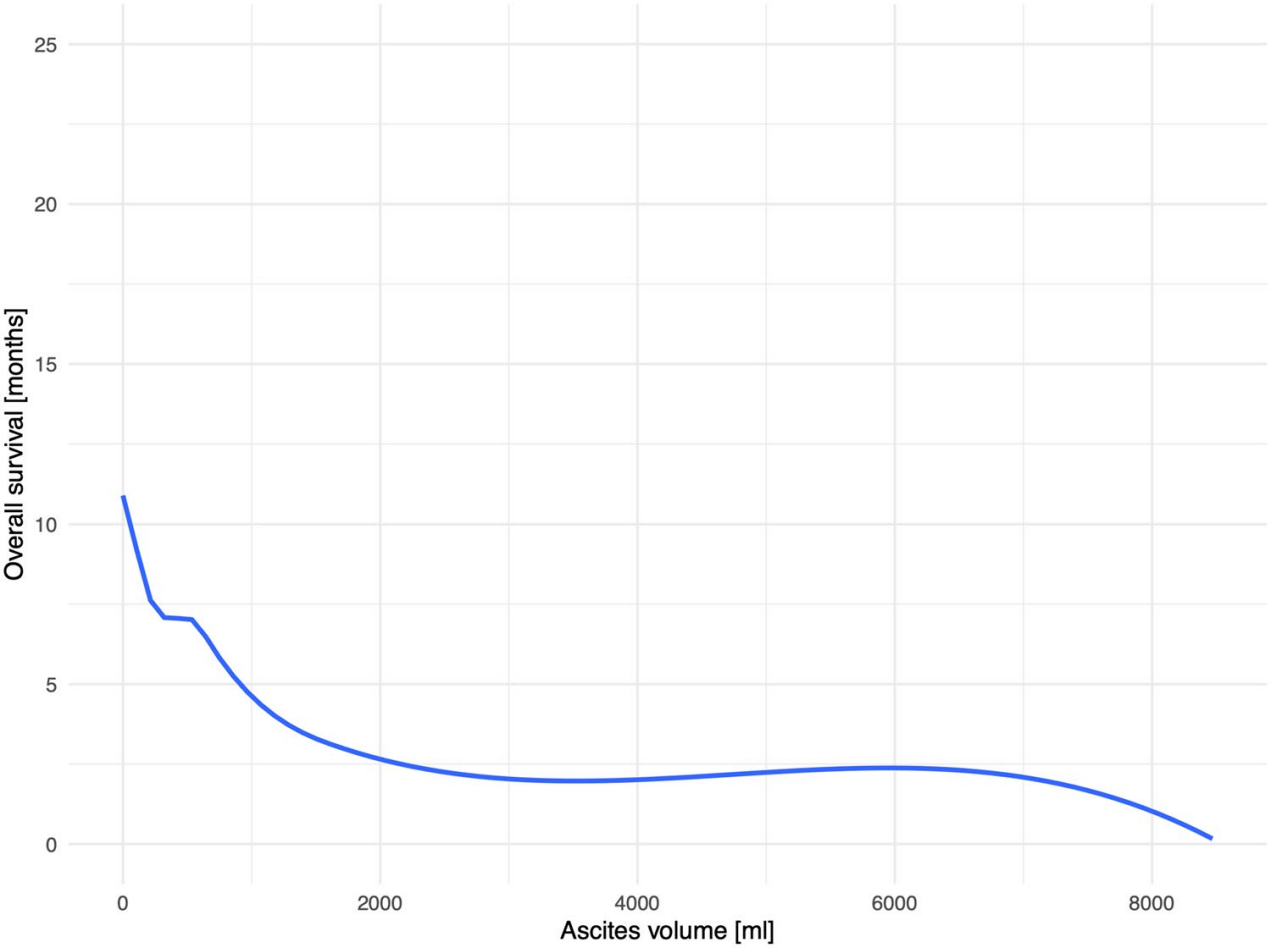
Correlation of ascites volume and overall survival.

Figure 3 illustrates the significant correlation (*p* < 0.001) of ascites volume and overall survival for the individual patients.

A univariate Cox hazard regression analyses identified ascites volume, apart from AFP, albumin, bilirubin, AST, tumor count, and tumor size, as significant prognostic factor (Table 3). None of the other included risk factors showed a significant association with OS. In the subsequent multivariate analysis, ascites volume remained an independent prognostic factor, as well as albumin, bilirubin, and AST.

**Table 3:** Univariate and multivariate Cox regression results of factors related to survival for all patients (n = 327).

Covariate	Category	Univariate			Multivariate		
		HR	95% CI	*p*-value	HR	95% CI	*p*-value
Age	Continuous	1.0	0.9–1.1	0.62			
AFP	Continuous	1.1	1.0–1.2	**0.04**	1.1	1.0–1.2	0.20
Albumin level	Continuous	0.6	0.5–0.6	** < 0.001**	0.7	0.6–0.8	** < 0.001**
Bilirubin level	Continuous	1.5	1.3–1.6	** < 0.001**	1.2	1.1–1.4	**0.005**
AST level	Continuous	1.4	1.2–1.5	** < 0.001**	1.2	1.1–1.4	**0.008**
ALT level	Continuous	1.0	0.8–1.1	0.43			
INR level	Continuous	1.1	1.0–1.2	0.34			
Platelet count	Continuous	1.1	0.9–1.2	0.41			
Tumor number	Continuous	1.2	1.0–1.3	**0.03**	1.0	0.9–1.2	0.93
Max. lesion size	Continuous	1.2	1.0–1.3	**0.04**	1.2	1.0–1.3	0.06
Ascites volume	Continuous	1.5	1.4–1.7	** < 0.001**	1.4	1.2–1.6	** < 0.001**

Significant values are in bold.

**Figure 4:**
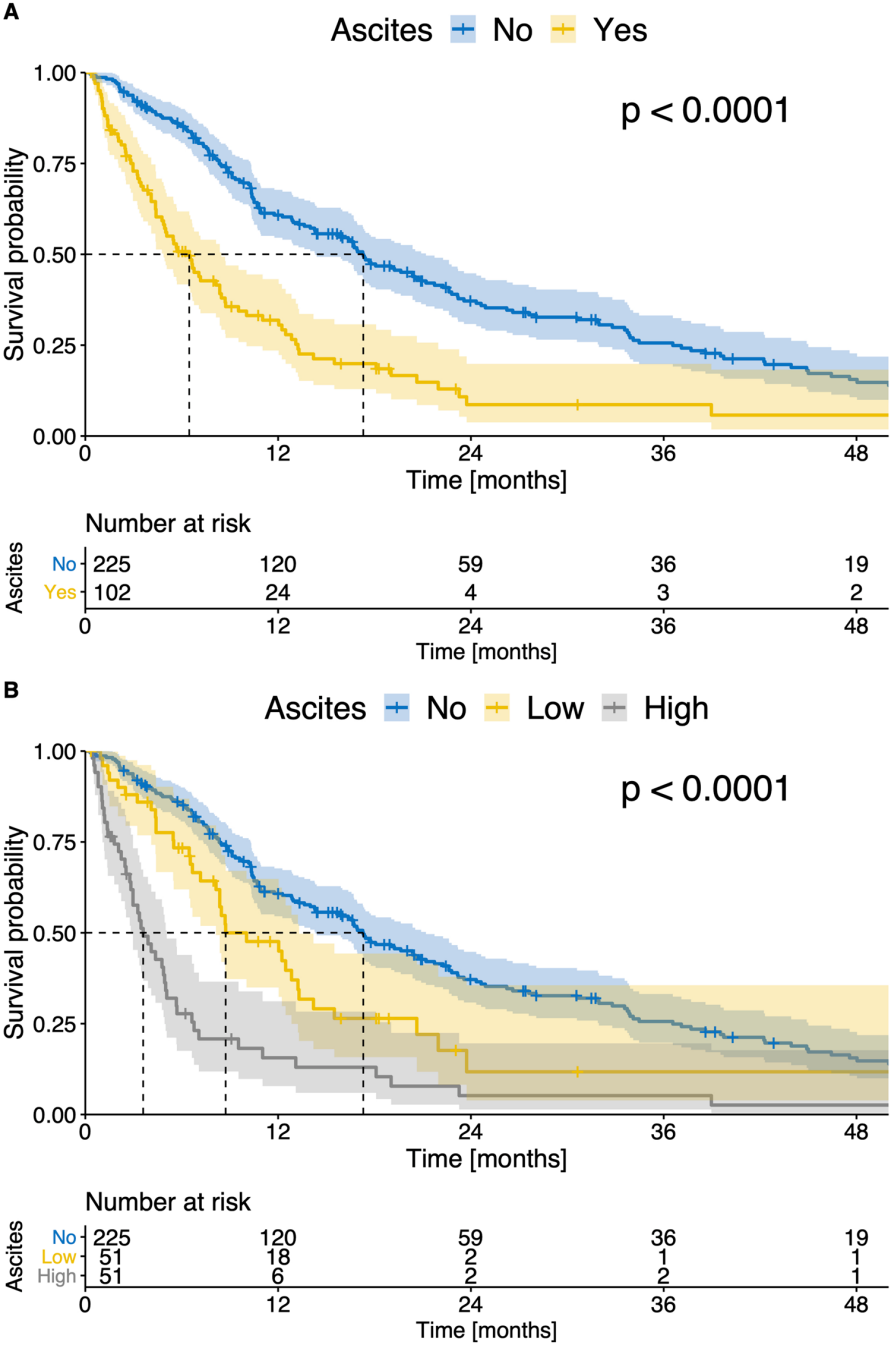
(**A**) Kaplan–Meier curves showing survival of patients with ascites and without ascites; (**B**) Kaplan–Meier curves showing survival of patients with no ascites, low ascites volume, and high ascites volume in all patients (n = 327).

When stratifying patients according to the presence of ascites, patients with ascites had a significantly impaired median OS compared to patients without ascites (6.4 vs 17.1 months, *p* < 0.001, Fig. 4A). In the next step, the patients were divided into a group with a low and a high ascites volume depending on the median (cut off 613.5 ml ascites). Patients with a high ascites volume had a significantly impaired survival (3.6 months) compared to patients with low ascites volume (8.6 months, *p* < 0.001, Fig. 4B). Patients with low ascites volume had a significantly impaired survival compared to patients with no ascites (*p* = 0.002, Fig. 4B).

When using the cut-off value of 300 ml previously suggested for patients with liver cirrhosis, patients with ascites above this value still had a significantly impaired survival compared to patients with ascites below this cut-off (4.0 vs 9.9 months, *p* < 0.001). Further, patients with ascites below a volume of 300 ml had a significantly impaired survival compared to patients without ascites (17.1 months, *p* = 0.02).

**Figure 5:**
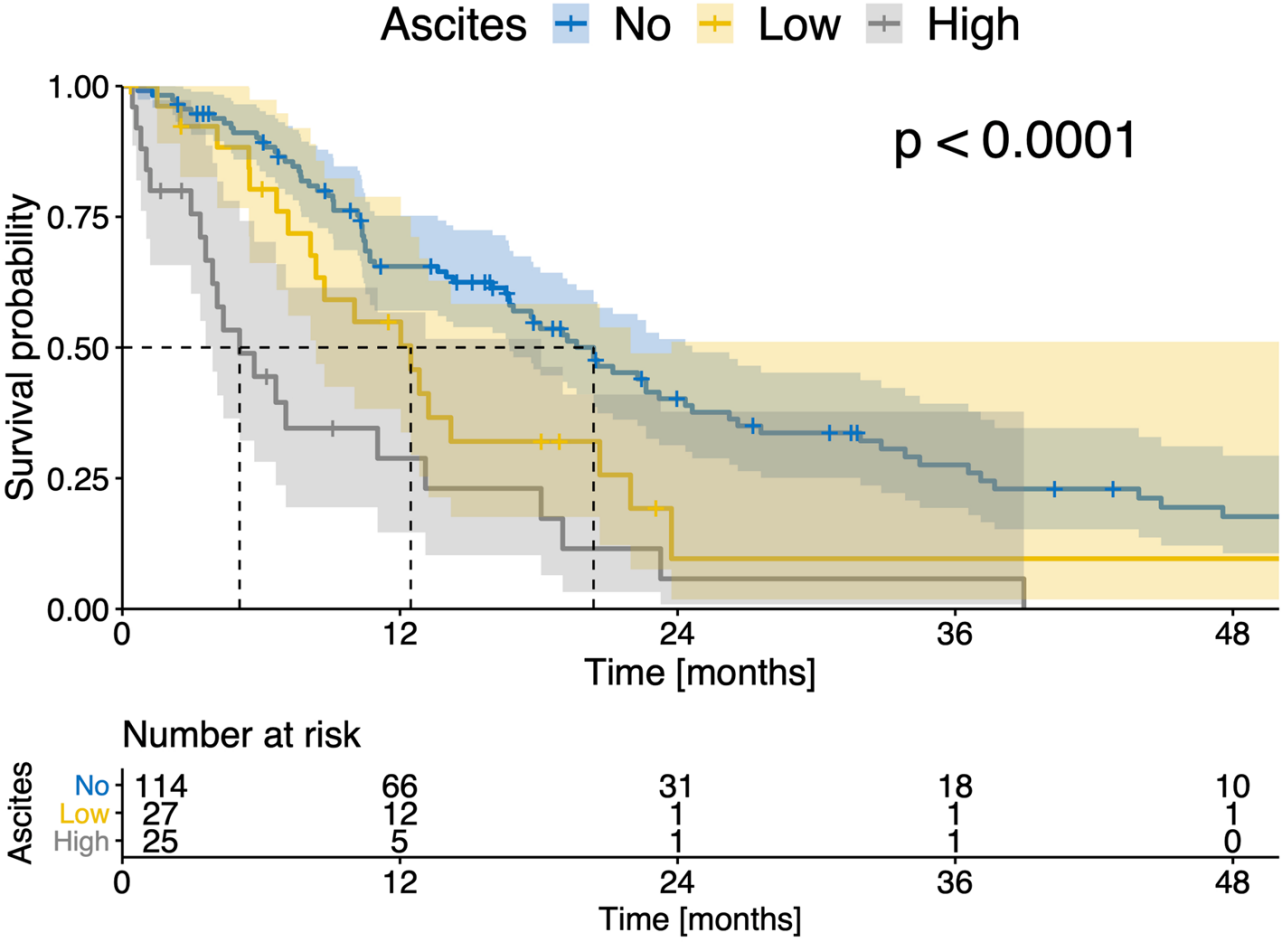
Kaplan–Meier survival curves show survival of patients with no ascites, low ascites volume and high ascites volume in the subgroup of patients within BCLC stage B (n = 166).

Subsequently, subgroup analysis was performed among 166 (50.8%) patients within BCLC stage B (i.e., the recommended TACE subgroup) yielding the same results ^3,4^: Patients with a high ascites volume had a significantly impaired survival (5.0 months) compared to patients with low ascites volume (12.3 months, *p* < 0.001, Fig. 5). Patients with low ascites volume had a significant impaired survival compared to patients with no ascites (20.1 months, *p* = 0.04, Fig. 5).

### Influence on subsequent treatment

The number of patients who were able to receive multiple TACE sessions was significantly lower in patients with ascites (n = 42/102 (41.2%) vs n = 183/225 (81.3%), *p* < 0.001). Among patients with ascites, the number of those who were able to receive multiple TACE session was significantly lower when their volume was above the median (n = 21/51 (41.2%) vs n = 39/51 (76.5%), *p* < 0.001). The median ascites volume of patients who were able to receive multiple TACE sessions was significantly lower (228 ml (IQR 26–944 ml) vs 954 ml (IQR 530–2670 ml), *p* < 0.001).

The number of patients who were able to receive a different subsequent treatment was significantly lower in patients with ascites (n = 12/102 (11.8%) vs n = 60/225 (26.7%), *p* = 0.002). Among patients with ascites, the number of those who were able to receive a different subsequent treatment was significantly lower when their volume was above the median (n = 1/51 (2.0%) vs n = 11/51 (21.6%), *p* = 0.003). The median ascites volume of patients who received subsequent treatment was also significantly lower (179 ml (IQR 30–448 ml) vs 660 ml (IQR 114–1605 ml), *p* = 0.04).

### Risk of hepatic decompensation

We further assessed the risk of an increase in the ALBI grade for patients who had an initial grade of 1 or 2 and an available follow-up ALBI value three months after TACE (n = 197/327 (60.2%)). Of these patients, a total of n = 61 (31.0%) patients had an ALBI grade increase three months after TACE. The relative number of patients who showed an increase in the ALBI grade was significantly higher in patients with ascites than without (n = 23/37 (62.2%) vs n = 38/160 (23.8%), *p* < 0.001). Notably, the majority of patients with ascites already had an initial ALBI grade of 3 (n = 57/102, 56%), therefore no further increase was possible. In the remaining subset of patients with ascites and an ALBI grade of 2, numbers were small with 23 patients showing an increase from grade 2–3 and 14 patients remaining at grade 2; in these small subsets the volume distributions did not differ significantly (*p* = 0.26).

## Discussion

In this study, we assessed the prognostic role of volumetric ascites quantification in patients with HCC undergoing TACE. We could show that the volume of ascites was associated both with poor survival outcome and with the likelihood to receive multiple TACE sessions.

While presence of ascites has been shown to be a prognostic factor for patients with HCC undergoing TACE treatment, combined therapy of TACE and apatinib or transarterial radioembolization, the literature regarding the influence of ascites quantification on OS in patients with HCC undergoing TACE is scarce ^11–13^. For patients with cirrhosis, stratification into groups without ascites, ≤ 300 ml, and > 300 ml has been previously proposed ^14^.

While our results also show that categorization into these volume categories result in a significant stratification regarding OS, in hazard regression analysis, the amount of ascites carried a significantly increased hazard ratio, underlining the importance of obtaining the true volume of ascites. Moreover, the volume of ascites was a significant predictor of OS in multivariate analysis when accounting for established tumor-related and liver function-related risk factors.

Until today, ascites is rarely quantified in clinical routine, due to the time-consuming nature of manual segmentations. Therefore, most scores rely on qualitative assessments, as employed in the Child–Pugh Score in sonography ^15^. In recent years, several semi-quantitative scores have been developed as well, counting for example the number of abdominal regions where free fluid can be detected ^16^.

Nowadays, organ segmentation and volume assessments can be performed by deep-learning algorithms. AI-based tools have the advantage that automated quantitative reports can be easily integrated into clinical workflows and can be automatically sent to the local hospital information system or image archiving and communication system ^17^. If commercial products are not yet available or their implementation is not feasible, there is an initial threshold to train and validate custom solutions, in terms of labeling the appropriate datasets, computation power, and software tools. However, the latter is reduced thanks to publicly available software packages and libraries ^18^.

In our cohort, free fluid was manually segmented on axial CT imaging. While this approach itself is not feasible in clinical routine, the data obtained this way forms the basis of a labeled training dataset, which will be used to train a deep-learning algorithm, more specifically a 3D-UNet, for automated ascites detection and segmentation at our institution, as has been done for other segmentation tasks including splenic volume assessment ^17^. Once trained, accurate ascites quantification will become available for other patient populations without further manual input.

In our study, ascites volume prior to TACE was lower in patients who were able to receive multiple TACE sessions. Therefore, ascites volume might serve as a quantifiable marker to assess TACE suitability in patients with unresectable HCC. With the advent of immunotherapy, the optimal time-point for a treatment switch in the concept of stage migration can be hard to identify; however, it is worth assessing as “an inappropriately high number of TACE sessions delays the switch to systemic therapy and may, in some cases, completely hinder the treatment switch due to the deterioration of liver function” ^19,20^.

Moreover, development of early ascites after TACE has been shown to be associated with impaired prognosis ^21^. This is in line with prior findings that have linked repeated TACE to an increase in portal hypertension and that portal hypertension is a predictor for adverse outcome in patients with liver cirrhosis and HCC ^22–24^.

Thus, we believe that volumetric ascites quantification as opposed to visual or semi-quantitative assessments will become part of the routine work-up for cirrhotic patients and patients with liver tumors in general and for patients with HCC in particular, given the concomitant occurrence of an aggressive tumor entity and an underlying liver disease. In our cohort, true ascites volume was an additional parameter to identify patients with poor survival and those likely to receive multiple TACE treatments. However, future largescale multicentric evaluations are needed to determine standard reference or cut-off values for this currently underused parameter.

The present study had several limitations. First, it was performed at a single-center and in a retrospective manner. However, the sample size was comparative to previous studies on this topic ^14^. Additionally, our dataset was well investigated and we only included patients with complete clinical, laboratory, and imaging data. To avoid a time bias, only patients from 2010 and later were included. These criteria minimized differences in the diagnosis and treatment decisions, which provided a more homogeneous study cohort.

Moreover, similar to the BCLC classification in which the Child–Pugh Score is incorporated at initial diagnosis to give recommendations for treatment and survival estimates, ascites was volumetrically assessed in our study at initial diagnosis as well. Therefore, management of ascites in the course of disease like medication or paracentesis was not taken into account.

Another limitation is that ascites due to portal hypertension and/or peritoneal carcinomatosis may have different prognostic relevance. However, since only one patient in our study had peritoneal metastases, this could not be further investigated in this study.

Furthermore, patients that underwent previous treatments were excluded. Second, we included patients that underwent either conventional or drug-eluting bead-delivered TACE. However, several previous studies have shown OS was not influenced by the TACE delivery technique ^25–27^.

Thus, we concluded that ascites in patients with HCC undergoing TACE is associated with a poor prognosis. Our results show that not only the presence but also the amount of ascites is highly relevant. Therefore, once automated software solutions are available, true ascites volume is likely to impact clinical decision making and to overcome its status of currently underappreciated imaging biomarker.

## Methods

The Ethics Committee of the Medical Association of Rhineland Palatinate, Mainz, Germany approved this study and waived requirement for informed consent for the retrospective analysis of clinical data (permit number 2021–15,984). This study was conducted following the ethical principles of the Declaration of Helsinki. Patient records and information were anonymized prior to analysis.

### Patients

A total of 714 patients with confirmed HCC that received TACE treatment in our tertiary care center between January 2010 and November 2020 were identified. Of these, 327 patients fulfilled the following inclusion criteria: (1) age above 18 years; (2) histologically or image-derived HCC diagnosis based on the EASL criteria; (3) no treatment performed prior to TACE; (4) no liver transplantation or tumor resection during the follow-up period after TACE; (5) pre-interventional contrast-enhanced CT scan for ascites volume assessment; (6) full availability of clinical, laboratory, and imaging data. A total of 387 patients were excluded, due to reasons shown in Fig. 1, and 327 treatment-naïve patients finally included.

### Diagnosis, treatment, and follow-up

HCC was diagnosed based on histological or image-derived criteria established by the EASL ^3,28^. All patients underwent contrast-enhanced CT for treatment planning. Indications for TACE were discussed in an interdisciplinary tumor board, which included hepatologists/oncologists, diagnostic and interventional radiologists, visceral surgeons, pathologists, and radiation therapists. TACE was performed in a standardized manner as previously described ^29,30^. Follow-ups included cross-sectional imaging, a clinical examination, and blood sampling. Follow-ups were performed every six or twelve weeks, depending on the presence of viable tumor tissue ^28^. Radiologic response was assessed using mRECIST criteria ^3,31^. The primary endpoint was the median overall survival (OS), defined as the interval between the initial TACE session and the date of death or last follow-up. Moreover, we investigated hepatic decompensation after TACE, which was objectively defined as an increase of the ALBI grade three months after the initial TACE as previously proposed ^32,33^.

### Data acquisition

The dataset was acquired from the clinical registry unit established at our tertiary care center, as previously reported ^28^. This dedicated, prospectively populated database contained data on all patients with primary liver cancer ^34^. Additional imaging and laboratory data were acquired from the radiology information system and the laboratory database. The final dataset included all available data on patient demographics, clinical assessments of the underlying liver disease and tumor, imaging, factors related to the TACE treatment, and laboratory parameters measured prior to the initial TACE treatment.

### Ascites volume assessment

In a first step, all cases were reviewed for the presence of ascites in our Picture Archiving and Communication System (Sectra, Linköping, Sweden). We extracted the portal venous phase of the abdominal CT scans for all patients with ascites in our dataset. Afterwards, ascites was manually segmented with the freely available LIFEx software (www.lifexsoft.org) ^35^. Two exemplary cases of ascites segmentation can be found in the supplement (Supplementary Figs. 1 and 2).

### Statistical analysis

All statistical analyses and graphics were performed in R studio (RStudio Team [2020]. RStudio: Integrated Development for R. RStudio, PBC, http://www.rstudio.com, last accessed 30 November 2023) with R 4.0.3 (A Language and Environment for Statistical Computing, R Foundation for Statistical Computing, http://www.R-project.org; last accessed 30 November 2023). Binary and categorical baseline parameters are expressed as absolute numbers and percentages. Continuous data are expressed as the median and range. Subgroups were compared with the Chi-Square test and Mann–Whitney U-test. Spearman’s rank correlation coefficient was used to assess the correlation between ascites volume and surrogates of liver function and tumor burden. Survival analyses were performed with the packages “survminer” and “survival” (https://cran.r-project.org/package=survminer, https://CRAN.R-project.org/package=survival, last accessed 30 November 2023). Survival was evaluated with Kaplan–Meier curves, and strata were compared with log-rank testing. We built univariate and multivariate Cox proportional hazards regression models and assessed hazard ratios (HRs) and the corresponding 95% confidence intervals (CIs). Prior to this, all continuous variables were normalized with the “scale” function (subtracting the mean and dividing by the standard deviation) in R. *P*-values < 0.05 were considered statistically significant.

### Ethics approval and consent to participate

The ethics committee of the Medical Association of Rhineland Palatinate, Mainz, Germany, approved this study and waived requirement for informed consent due to the retrospective nature of the study (permit number 2021–15984).

### Supplementary Information

Supplementary Figure 1.

Supplementary Figure 2.

## Data Availability

Data cannot be shared publicly because of institutional and national data policy restrictions since the data contain potentially identifying patient information. Data are available upon request from the Johannes Gutenberg University Mainz Medical Center (contact via radiologie-sekretariat@unimedizinmainz.de) for researchers who meet the criteria for access to confidential data (please provide the manuscript title with your enquiry).

## Abbreviations

AFP: Alpha-fetoprotein
BCLC: Barcelona clinic liver cancer
EASL: European Association for the study of the liver
Cis: Confidence intervals
HCC: Hepatocellular carcinoma
HRs: Hazard ratios
INR: International normalized ratio
IQR: Interquartile range
OS: Overall survival
TACE: Transarterial chemoembolization
