# Supplementary figures and images for: Amount of ascites impacts survival in patients with hepatocellular carcinoma undergoing transarterial chemoembolization advocating for volumetric assessment
Source: Sci Rep. 2024 Jul 17;14:16550. doi: 10.1038/s41598-024-67312-2 (PMC11255265; doi:10.1038/s41598-024-67312-2)

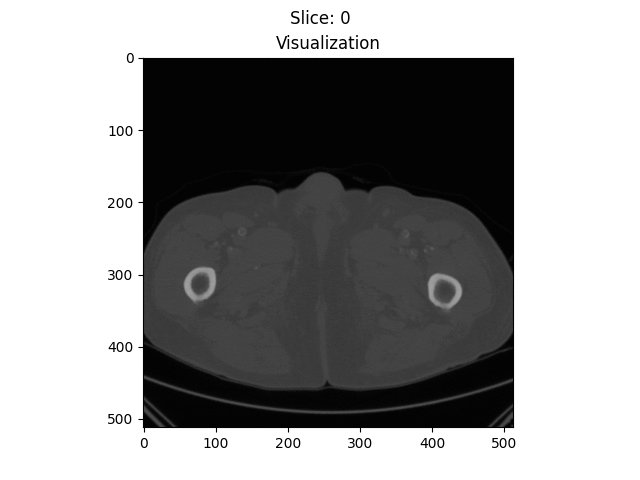

Supplement: Supplementary file 1 — Supplementary Figure 1. [file 41598_2024_67312_MOESM1_ESM.gif]

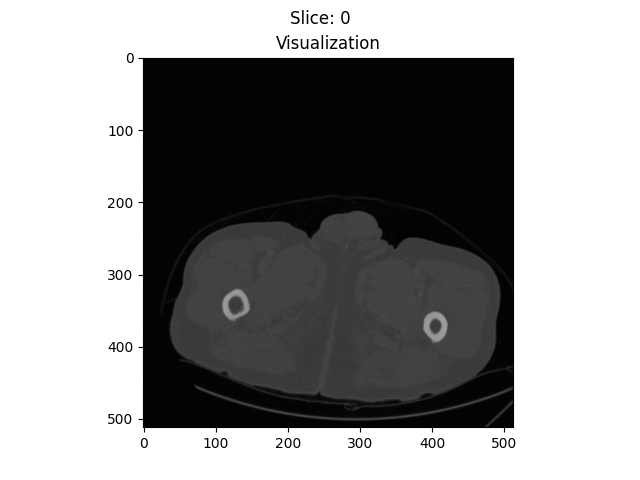

Supplement: Supplementary file 2 — Supplementary Figure 2. [file 41598_2024_67312_MOESM2_ESM.gif]
